# Supplementary material for: The Impacts of Antivirals on the Coronavirus Genome Structure and Subsequent Pathogenicity, Virus Fitness and Antiviral Design
Source: Biomedicines. 2020 Sep 24;8(10):376. doi: 10.3390/biomedicines8100376 (PMC7601523; doi:10.3390/biomedicines8100376)
Supplement: Supplementary file 1 [file biomedicines-08-00376-s001.pdf]

Supplementary Materials

The impacts of antivirals on the coronavirus genome structure and subsequent pathogenicity, virus fitness and antiviral design

Ching-Hung Lin[1], Cheng-Yao Yang[1], Shan-Chia Ou[2], Meilin Wang[3], Chen-Yu Lo[1], Tsung-Lin Tsai[1], Hung-Yi Wu[1]*

1 Graduate Institute of Veterinary Pathobiology, College of Veterinary Medicine, National Chung Hsing University, Taichung 40227, Taiwan; tw23whale@hotmail.com (C.-H.L.); yangchengyao@nchu.edu.tw (C.-Y. Y); axfbji7917@gmail.com (C.-Y.L.); windtaker10@msn.com (T.-L.T.)

2 Graduate Institute of Microbiology and Public Health, College of Veterinary Medicine, National Chung Hsing University, Taichung 40227, Taiwan; scou@dragon.nchu.edu.tw (S.-C.O.)

3 Department of Microbiology and Immunology, School of Medicine, Chung-Shan Medical University and Clinical Laboratory, Chung-Shan Medical University Hospital, Taichung 40201, Taiwan ; wml@csmu.edu.tw (M. W.)

* Correspondence; E-Mail: hwu2@dragon.nchu.edu.tw (H.-Y.W.); Telephone: +886-4-22840369; Fax: +886-4-22862073

Supplementary Figures

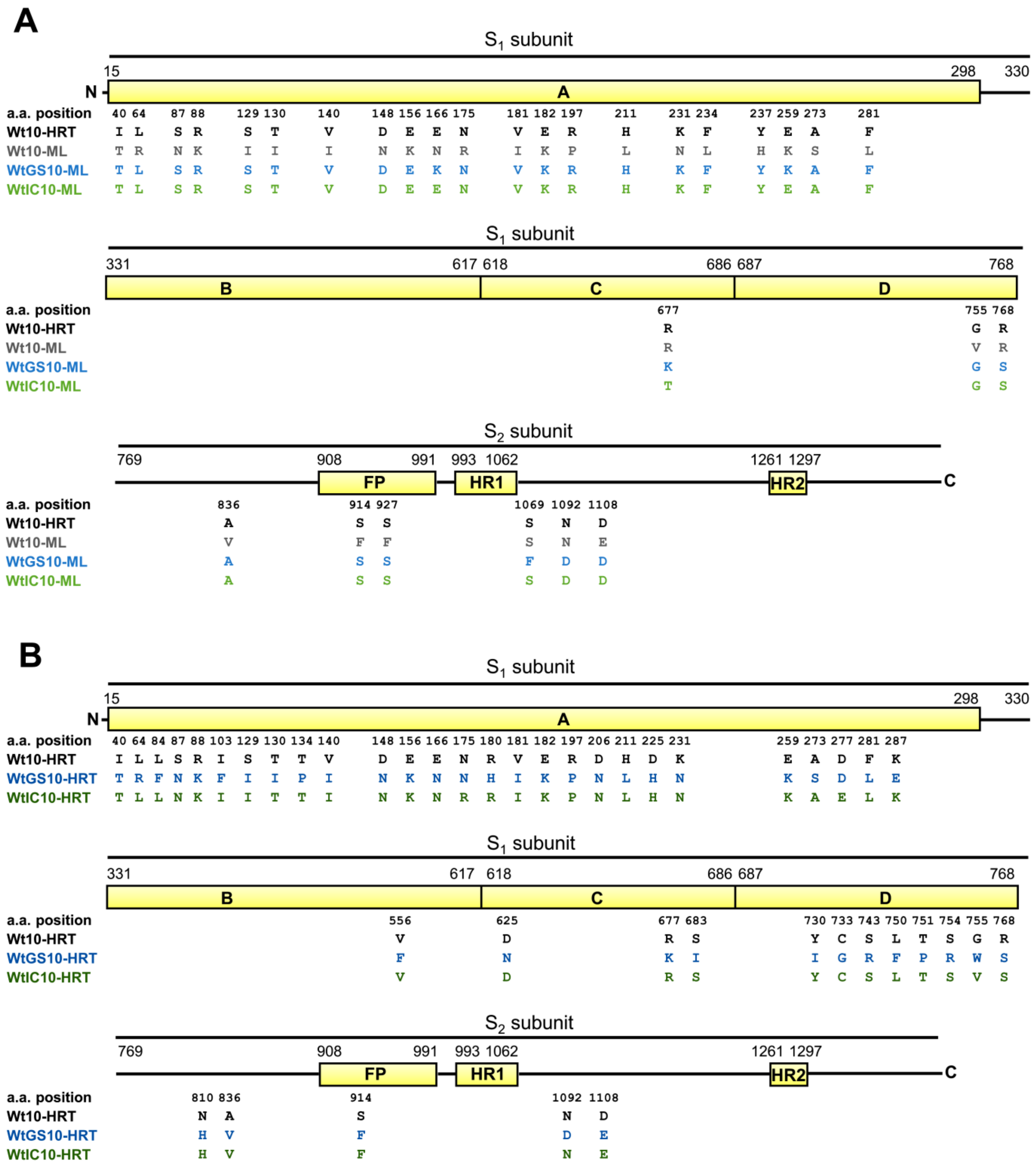

Figure S1. Comparison of the S protein aa sequences identified from HRT-18 cells and ML cells infected with wt BCoV and its variants. (A) Linear schematic of BCoV S protein showing the comparison of the aa sequences for Wt10-ML, WtGS10-ML and WtIC10-ML identified from ML cells infected with Wt10, WtGS10 and WtIC10, respectively, at VP10. **(B)** Linear schematic of BCoV S protein showing the comparison of the aa sequences identified from HRT-18 cells under the treatments of antiviral GS-5734 (WtGS10-HRT, i.e., WtGS10 in Figure 1) and poly IC-induced innate immunity (WtIC10-HRT, i.e., WtIC10 in Figure 4) at VP10. Wt10-HRT (i.e., Wt10 in Figures 1 and 4): Viral RNA collected from HRT-18 cells infected with wt BCoV at VP10.
